# Supplementary material for: In Situ Structures of the Ultra-Long Extended and Contracted Tail of Myoviridae Phage P1
Source: Viruses. 2023 May 29;15(6):1267. doi: 10.3390/v15061267 (PMC10304247; doi:10.3390/v15061267)
Supplement: Supplementary file 1 [file viruses-15-01267-s001.zip › Supplementary Figures and Table.pdf]

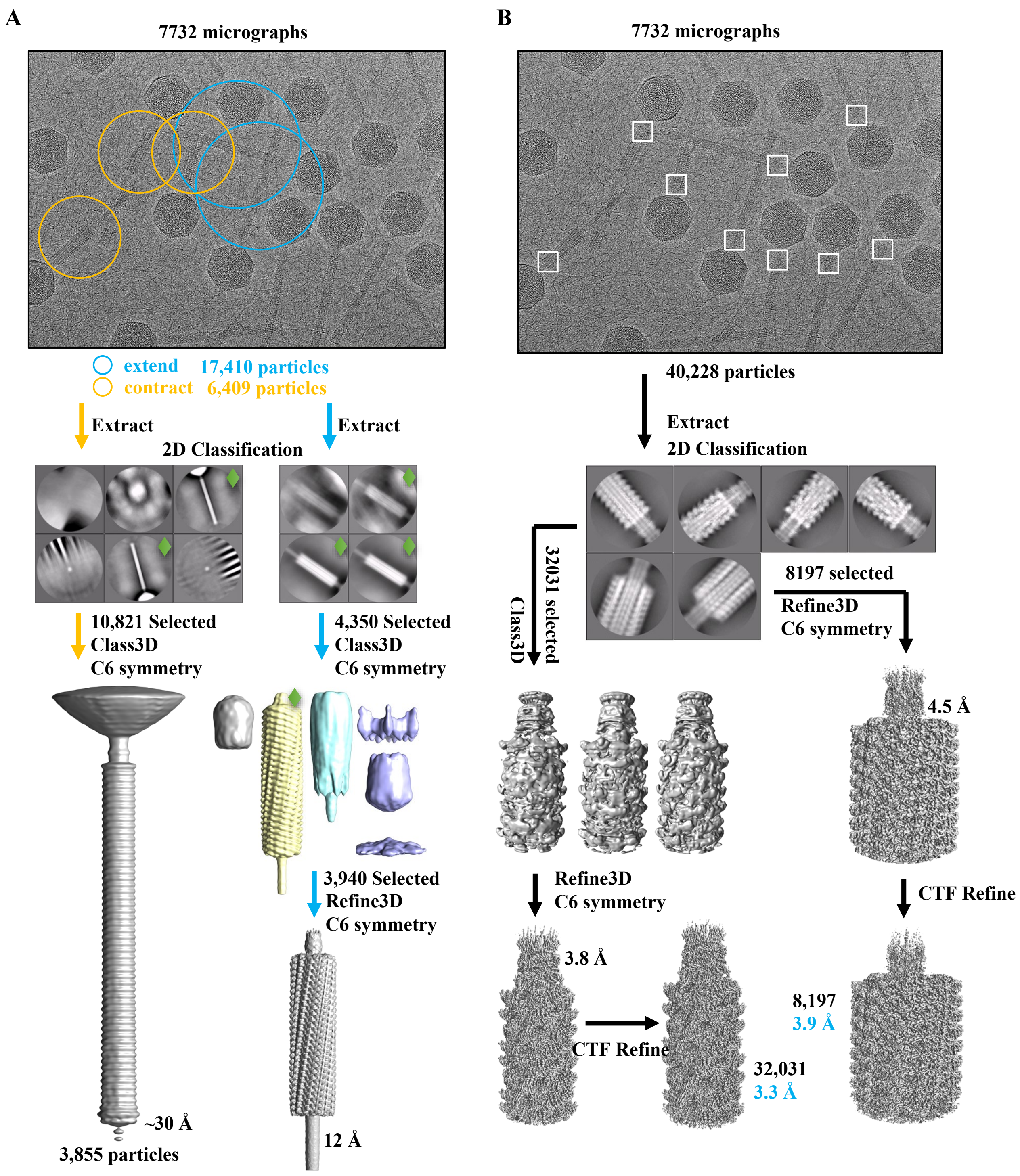

Figure S1. Workflows for the tail structure determinations of the extended and contracted states. (**A**) Intact tail data processing, including manual particle, 2D classification, 3D classification and refinement. (**B**) Local reconstruction data processing, including manual picking particles, 2D classification, 3D classification and refinement, CTF refinement.

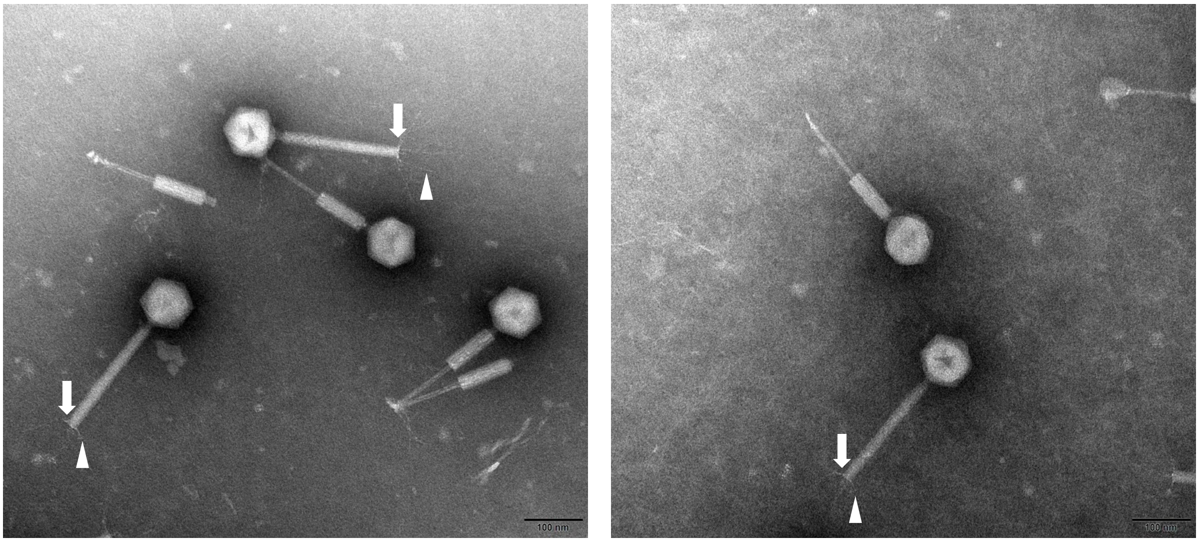

Figure S2. Negative-stain images of phage P1 showing the missing of baseplate in the contracted tail. The baseplate and fiber in the extended tails are marked with white arrows and white triangles, respectively.

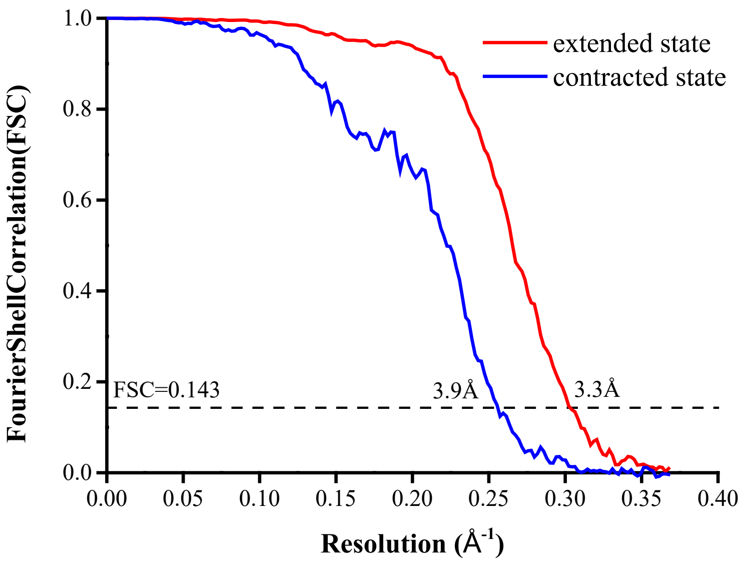

Figure S3. Fourier shell correlation (FSC) curves of the density map in the extended (red) and contracted (blue) states generated by the RELION program.

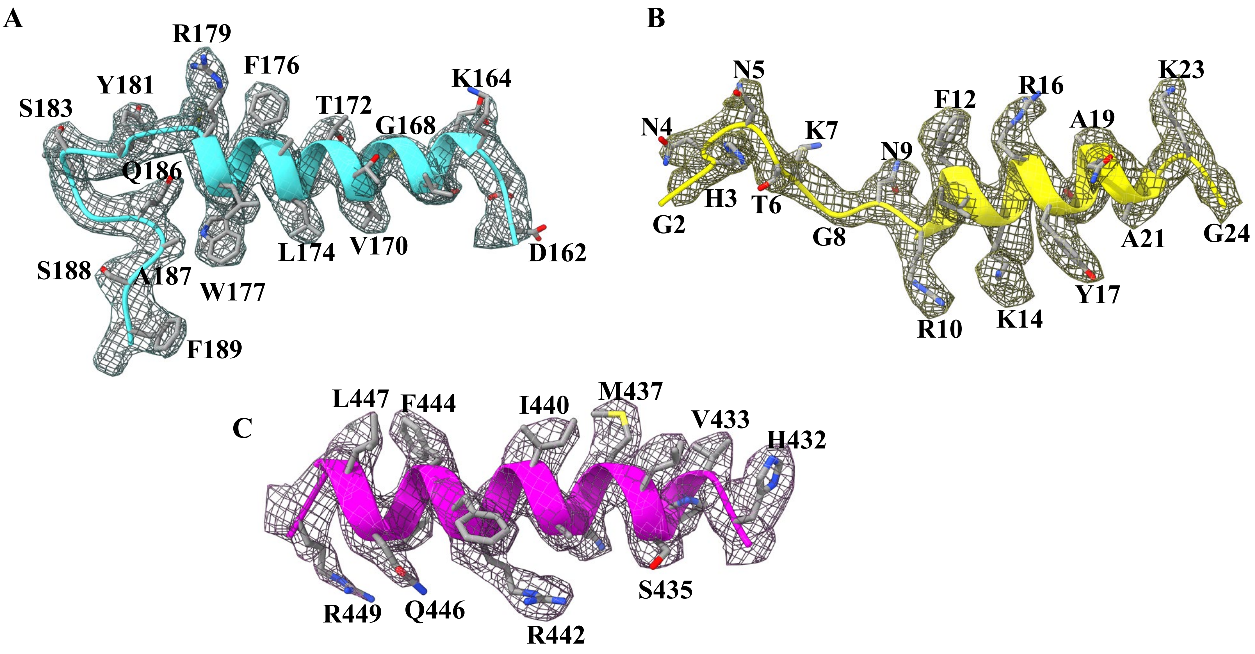

Figure S4. Representative density maps (mesh) of the proteins gp24 (A), BplB (B), gp22 (C) superimposed on their atomic models.

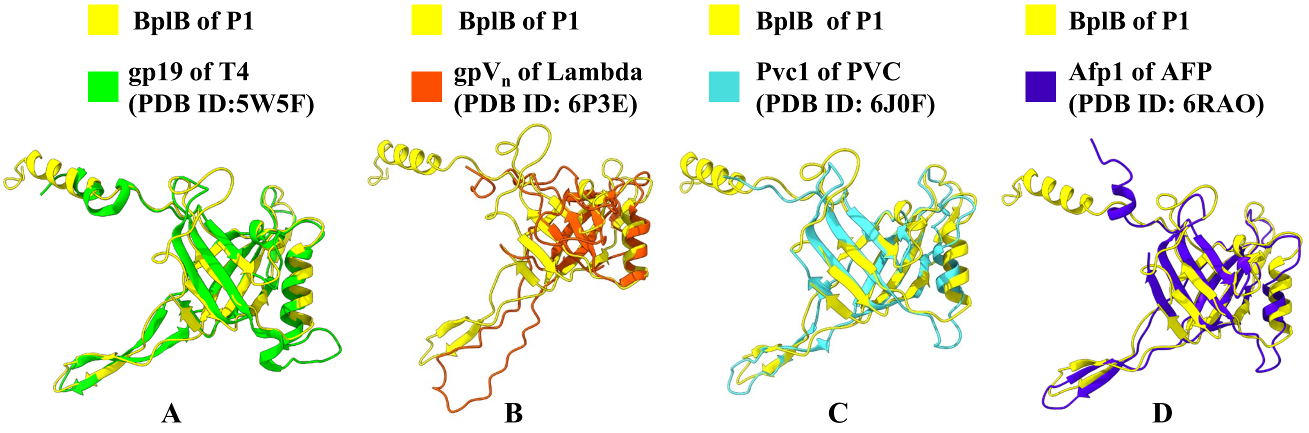

Figure S5. Structural superpositions of the tube protein BplB of P1 and the homologous proteins of phage T4 (A), phage lambda gpV$_n$ (B), PVC (C), and AFP (D).

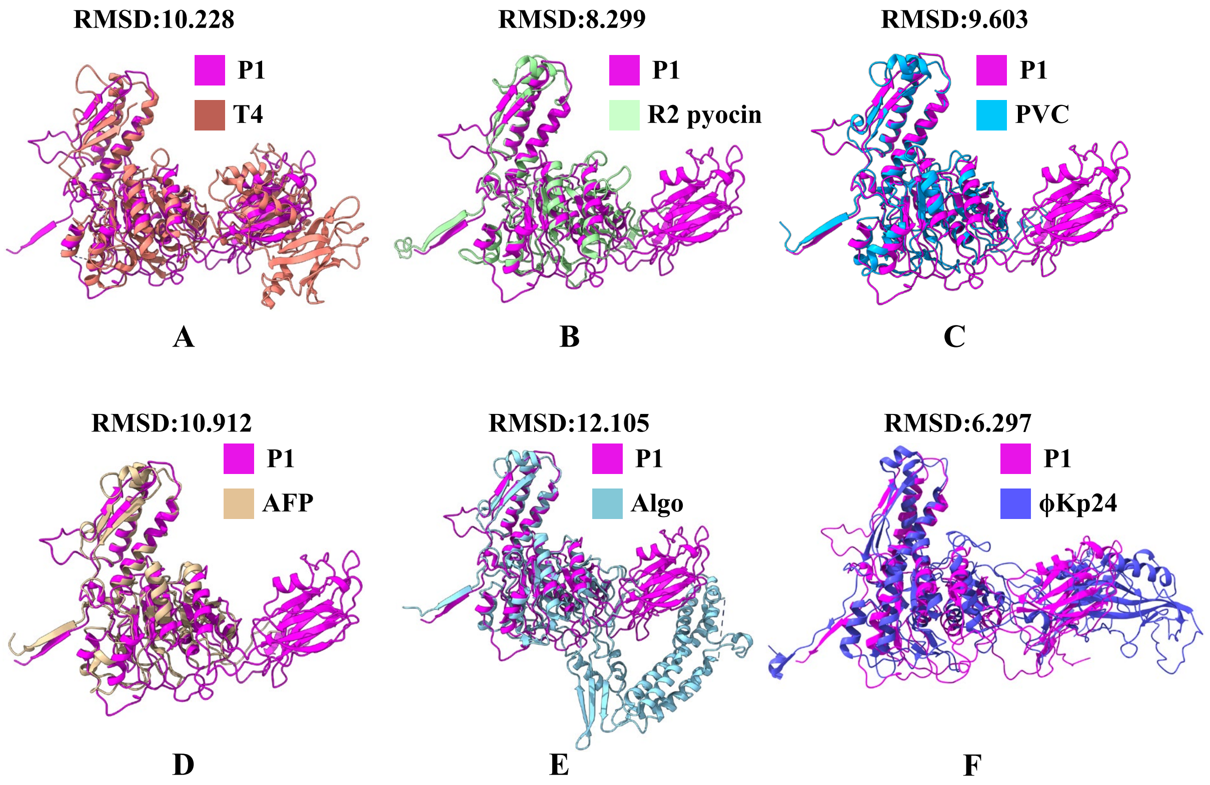

Figure S6. Structural superpositions of the sheath protein gp22 of P1 and the homologous proteins of phage T4 (PDB ID: 3J2N) (A), R2 pyocin (PDB ID: 3J9Q) (B), PVC (PDB ID: 6J0F) (C), AFP (PDB ID: 6RAO) (D), Algo (PDB ID: 7AE0) (E), and ϕKp24 (PDB ID: 8AU1) (F). The RMSD of domain I and domain II between the structural superposition is labeled.

Table S1. Cryo-EM data collection and model statistics.

DATA Collection				
**Electron microscopy**	Titan Krios G3i			
**Pixel size (Å)**	1.36			
**Total micrographs**	7732			
**Electron exposure (e/Å^2)**	30			
**Voltage (KV)**	300			
**Cs**	0			
**Defocus range**	-1.6 ~ -2.2 µm			
**Map Refinement**				
**Map**	extended			contracted
**Map resolution(Å)**	3.3			3.9
**Final particles**	32031			8197
**B-factor**	80			80
**EMDB-ID**	EMD-36130			EMD-36127
**Atomic model Refinement**				
	extended			contracted
**protein**	gp24	gp22	BplB	gp22
**PDB-ID**	8JAN			8JAJ
**CC (model to map fit)**	0.8661			0.8312
**Rama Favored (%)**	95.75			96.56
**Rama Allowed (%)**	4.25			3.24
**Rama Outliers (%)**	0.00			0.19
**MolProbity score**	1.5			1.56
**Clashscore**	4.26			6.08
**Poor rotamers (%)**	0.00			0.00
